# Seven classes of antiviral agents

**DOI:** 10.1007/s00018-022-04635-1

**Published:** 2022-11-27

**Authors:** Aleksandr Ianevski, Shahzaib Ahmad, Kraipit Anunnitipat, Valentyn Oksenych, Eva Zusinaite, Tanel Tenson, Magnar Bjørås, Denis E. Kainov

**Affiliations:** 1 Department of Clinical and Molecular Medicine (IKOM), Norwegian University of Science and Technology, 7028 Trondheim, Norway; 2 Institute of Technology, University of Tartu, 50411 Tartu, Estonia; 3 Institute for Molecular Medicine Finland, University of Helsinki, 00014 Helsinki, Finland

**Keywords:** Virus, Virus–host interaction, Antiviral, Broad-spectrum antiviral, Antiviral drug combination

## Abstract

The viral epidemics and pandemics have stimulated the development of known and the discovery of novel antiviral agents. About a hundred mono- and combination antiviral drugs have been already approved, whereas thousands are in development. Here, we briefly reviewed 7 classes of antiviral agents: neutralizing antibodies, neutralizing recombinant soluble human receptors, antiviral CRISPR/Cas systems, interferons, antiviral peptides, antiviral nucleic acid polymers, and antiviral small molecules. Interferons and some small molecules alone or in combinations possess broad-spectrum antiviral activity, which could be beneficial for treatment of emerging and re-emerging viral infections*.*

**Supplementary Information:**

The online version contains supplementary material available at 10.1007/s00018-022-04635-1.

## Background

The COVID-19 pandemic associated with the severe acute respiratory syndrome coronavirus 2 virus (SARS-CoV-2) has stimulated the development of known and the discovery of new antiviral measures. According to CDC and WHO, protective clothing, face masks, washing hands, cleaning surfaces with detergents, social distancing, travel restrictions, and isolating the infected people have all helped to limit the spread of the respiratory virus. Vaccination has improved population protection from severe SARS-CoV-2 infection [1]. Antiviral agents have also provided prophylactic and therapeutic protection against the infection [2]. Here, we review antiviral agents and specifically focus on broad-spectrum antivirals and their combinations with other agents, because these options could help us to better prepare for the next viral epidemics and pandemics and reduce morbidity and mortality from viral diseases, increase healthy life expectancy, and improve quality of our lives.

## Antiviral agents and therapies

### Targeting virus replication

Most viruses are recognized as hormones, cytokines or nutrient sources by one or more receptors on host cell surface. This mimicry strategy allows viruses to attach to specific cells, cross the plasma membrane barrier, enter the cell, and access essential cellular machineries [3]. Inside the cells, host or viral polymerases amplify viral genomes through RNA or DNA intermediates. Host or viral RNA polymerases also transcribe viral genes into mRNAs, which are translated on host ribosomes into viral proteins. Some viruses require host cell membranes for replication and/or assembly of new viral particles.

In addition, many viruses require other host factors for their efficient replication. Analysis of virus–host interactions revealed the critical nodes of the molecular networks and factors (such as underlying diseases, their interventions with commonly prescribed drugs, diets, etc.) that can affect the interactions [4]. Some nodes are unique, while others are similar/common for different viruses.

**Fig. 1:**
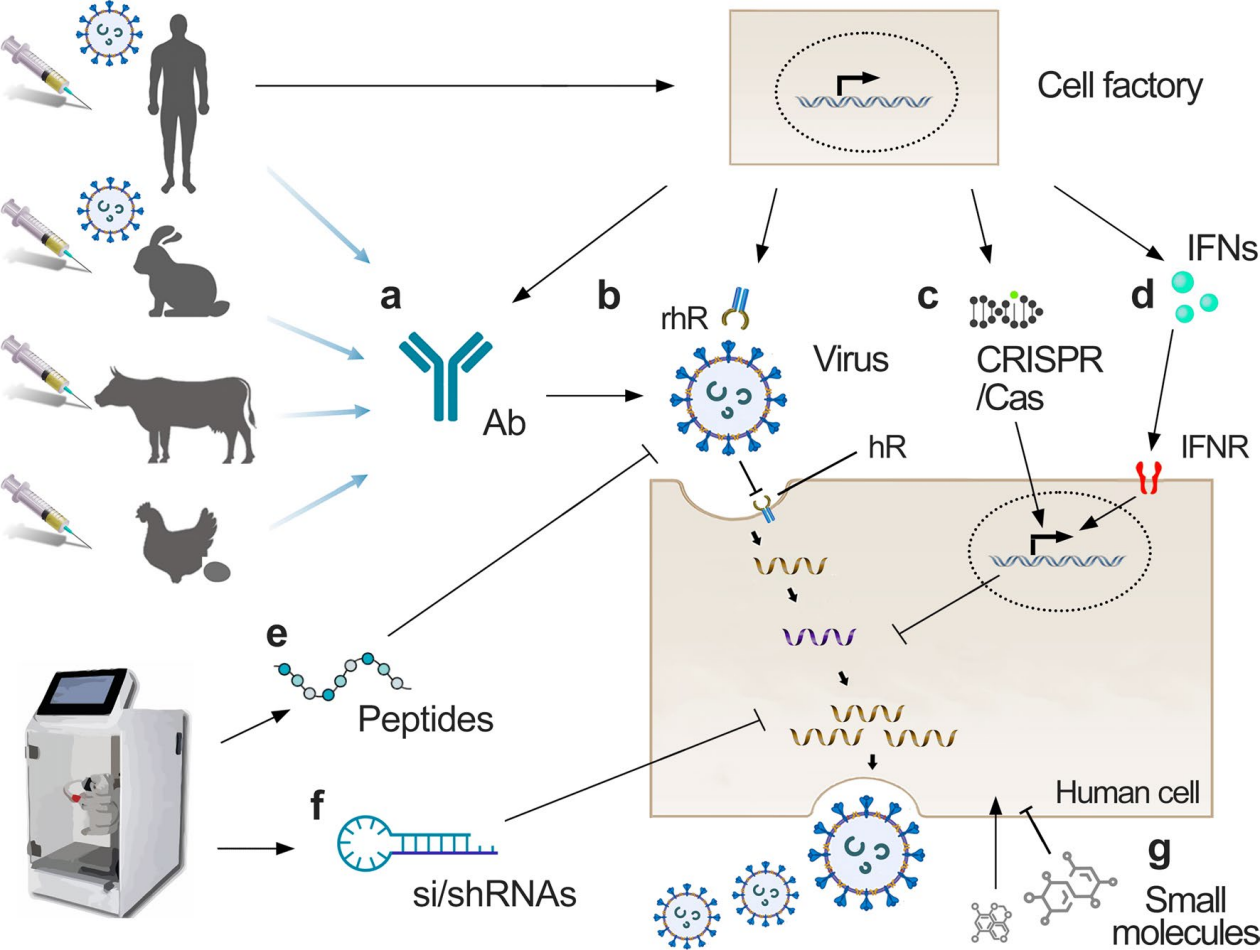
Antivirals, their potential sources, and stages of virus replication they affect (where appropriate). **a** Neutralizing antibodies. **b** Neutralizing recombinant soluble human receptors. **c** Antiviral CRISPR/Cas systems. **d** Interferons. **e** Antiviral peptides. **f** Antiviral nucleic acid polymers, including small interfering (si)/small hairpin (sh)RNAs. **g** Antiviral small molecules

In a recent year, researchers identified many antiviral agents that target the common and unique nodes and prevent viruses from interacting with the host. Figure 1 depicts different antivirals that target virus or host factors, or their interactions.

### Antiviral agents

Antivirals can be divided based on the targets which can be either host or viral factors. Virus-directed agents bind viral proteins or nucleic acids involved in viral entry, transcription, replication of the viral genome, assembly, and release of infectious viral particles. Host-directed antivirals modulate the activity of host factors and pathways involved in the synthesis, processing, and transport of viral building blocks, as well as in the development of antiviral and inflammatory responses. It should be noted that host-directed agents may simultaneously target multiple steps of viral replication. To date, there is no database summarizing all existing antivirals. However, some approved, investigational, experimental, and abandoned antivirals can be found in various databases, e.g., DrugVirus.Info [5], FluDB, DrugBank, and AVPdb. Antiviral agents are presented in numerous molecular forms, including small molecules, peptides, neutralizing antibodies, interferons (IFNs), CRISPR-Cas systems, si/shRNA, and other nucleic acid polymers (NAPs), which are described in corresponding subsections below.

#### Virus-neutralizing antibodies

Virus-neutralizing antibodies (nAbs) stick to the viral surface proteins and stop virus from getting inside our cells (Fig. 1a). nAbs also signal to immune cells to come and help destroy the virus. There are different sources for production of nAbs:

*Human-derived nAbs*. Human convalescent plasma can be collected from vaccinated individuals or patients who have recently recovered from the relevant viral disease [6]. Only some plasma samples contain high titers of nAbs [7]. The plasma can be used to transfer passive antibody immunity to those who have recently been infected or have yet to be exposed to the virus. However, the benefits of convalescent plasma for the treatment of viral infections are uncertain [8]. Peripheral blood mononuclear cells (PBMCs) can be also collected from recently recovered or vaccinated individuals. After identification of the serological responses to viral antigens, antibody genes of B-cells can be cloned, and nAbs can be identified and purified in large quantities [9, 10]. Such antibodies could have prophylactic or therapeutic benefits [11]. However, in some cases Abs can enhance virus infection and contribute the pathogenesis of viral diseases [12, 13].

*nAbs derived from small laboratory mammals*. When it is difficult to obtain blood samples from infected or vaccinated people, or when a virus of interest is highly pathogenic/virulent, small laboratory mammals could serve as a source for production of nAbs under appropriate safety conditions. For example, rabbits can be infected or immunized with a virus or its components, respectively. In case of vaccination, the production of antibodies can be boosted with second dose of antigene. Binding assays, or ELISA-based human receptor blocking assay, can be performed to screen and select nAb clones. The antibodies are then humanized, i.e., rabbit antibody constant regions are replaced with human antibody constant regions. And the amino acids in Complementarity-determining regions (CDR) and framework regions of the variable domains of rabbit Abs are substituted with amino acids of closest germlines and known human antibody sequences. The resulting humanized nAbs can be used as a therapeutic agent in humans [14].

*Colostrum-derived nAbs*. Pregnant large farm mammals produce antibodies upon immunization, and the antibodies move into the colostrum immediately before delivery of offspring. Such polyclonal nAbs showed potential to serve as prophylactic agents against influenza and SARS-CoV-2 infection in vivo [15, 16].

*Chicken egg yolk-derived nAbs*. Chicken egg yolk polyclonal immunoglobulins (IgYs) are attractive targets for pre-clinical and clinical development for the rapid management of outbreaks of emerging and re-emerging viruses. They can be readily generated in large quantities using egg-laying hens. It was shown that IgYs neutralized SARS-CoV, SARS-CoV-2, influenza virus, Ebola virus, Zika virus, Dengue virus, and human norovirus in vitro and in animal models, and had favorable safety profiles in man. Similarly to mammalian IgGs, IgYs are fast-acting. By contrast to human IgGs, they can neither bind to receptors nor activate complement components in humans; therefore, the exacerbation of viral diseases through antibody-dependent enhancement could be potentially avoided [17, 18]. In addition, other animals could be used as production of broadly neutralizing polyclonal antibodies [19].

#### Recombinant human receptors as antivirals

While viruses may mutate and escape recognition by mAbs, they must maintain the capacity to bind their host receptors (hRs). It was shown that recombinant soluble human angiotensin-converting enzyme 2 (ACE2) receptor can block SARS-CoV-2 infection. Moreover, the rhACE2 was resistant to viral escape [20]. Thus, neutralizing biologics based on recombinant soluble hRs could represent an interesting avenue in antiviral drug development (Fig. 1b) [21].

#### CRISPR/Cas-based antiviral therapy

In bacteria, CRISPR/Cas systems are the adaptive immune systems that protect against invading bacteriophages and foreign nucleic acids [22]. The Cas9-, Cas12- (both cut dsDNA), and Cas13- (cuts ssRNA) based systems have been adapted for treatment of viral infections in vitro and in vivo in mammals (Fig. 1c) [23]. For example, EBT-101 was a first-in-human one-time gene therapy against HIV with adeno-associated virus used to deliver the CRISPR/Cas9 system [24]. CRISPR/Cas9 system can be also targeted to dsDNA virus genomes and impair their replication [25]. IAV, LSMV, VSV, SARS-CoV2, and other ssRNA virus infections can be detected and inhibited using CRISPR/Cas13 [26, 27]. However, such systems should be exploited more for chronic and latent viral infections.

#### Antiviral interferons

Interferons (IFNs) are a group of signaling proteins made and released by human cells in response to infection with several viruses causing degradation of viral nucleic acids in infected cells and triggering antiviral responses in nearby non-infected cells (Fig. 1d). IFNs are classified according to the cellular receptor to which they bind. Type I IFNs (IFN-alpha, IFN-beta, IFN-epsilon, IFN-kappa, and IFN-omega) bind to the IFNAR1/2, type II IFNs (IFN-gamma) bind to the IFNGR1/2, whereas type III IFNs (IFN-lambda-1-4) together with interleukin 10 receptor 2 activate the IFNL receptor. Recombinant human IFNs (rhIFNs) have been approved for treatment of hepatitis C virus (HCV) and hepatitis B virus (HBV) infections. Although rhIFNs are effective against a variety of other viruses, including coronoviruses, they possessed limited efficacy and can cause adverse effects in vivo [28–31].

#### Antiviral peptides

Antiviral peptides (AVP) are polymers that have been experimentally verified to interfere with virus replication (Fig. 1e). They can be divided into different categories according to their mechanisms of action, including binding/attachment inhibitors, fusion and entry inhibitors, viral enzyme inhibitors, virus assembly inhibitors, and peptides with indirect effects on the viruses. Such AVPs can be designed computationally based on the available structural information of virus proteins and chemically synthesized. Alternatively, they can be obtained from natural sources [32]. Some AVP sequences and their modes of action (MOAs) could be found in http://crdd.osdd.net/servers/avpdb/ server, which is manually curated open-source archive with experimentally verified 2683 AVPs, including 624 modified AVPs that target 60 medically relevant viruses [33]. Altogether, 144 antiviral peptides can be found in the “Antimicrobial Peptide Database (APD2)” [http://aps.unmc.edu/AP/main.php]. The AVPs can also serve as delivery vehicles for other antivirals.

#### Antiviral nucleic acid polymers, including si/shRNAs

Antiviral nucleic acid polymers (NAPs) can directly inhibit viral entry or replication by binding to the virus particle, its building blocks, or RNA/DNA replication intermediates (Fig. 1f). Several NAPs are under development for treatment of hepatitis C, influenza virus, norovirus, HSV, and HIV infections [34]. In particular, si/shRNAs can guide specific virus clearance via RNAi in mammalian cells [35, 36]. NAPs can be designed computationally, based on the available structural information of a virus, and chemically synthesized. Alternatively, they can be produced in situ or in vitro [37]. NAPs can also serve as delivery vehicles for other molecular forms of antivirals.

#### Small-molecule antivirals

Small-molecule or small molecular-weight antivirals attenuate viral replication (Fig. 1g). Some small molecules are derived from natural sources, whereas others are chemically synthesized [38, 39]. Some small molecules affect critical functions of viral factors, whereas others interfere with host factors and pathways necessary for virus replication. In addition, some small molecules modulate the development of antiviral and inflammatory responses [2, 40]. An interesting subclass of host-directed small molecules is pro-apoptotic agents that promote the death of infected cells without affecting uninfected cells [41–43]. By comparison to virus-directed antivirals, host-directed small molecules often have more side effects [44].

### Broad-spectrum antivirals

Antivirals can target one or more viruses. Agents that inhibit the replication of many viruses are called broad-spectrum antivirals (BSAs). BSAs that inhibit the replication of viruses from the same or different viral families are called intra- or inter-family antivirals, respectively (Supplementary data, Fig. 2). For example, IFN-alpha, IFN-beta, ribavirin, remdesivir, and favipiravir are inter-family, whereas minocycline (inhibiting Flaviviridae) and brivudine (inhibiting Herpesviridae) are intra-family BSAs. The DrugVirus.Info web server summarized more than 250 BSAs. It also allowed tracking of the progress of BSA development [5, 45]. Such information can be utilized for rapid identification of potential BSAs to combat emerging or re-emerging viral infections.

**Fig. 2:**
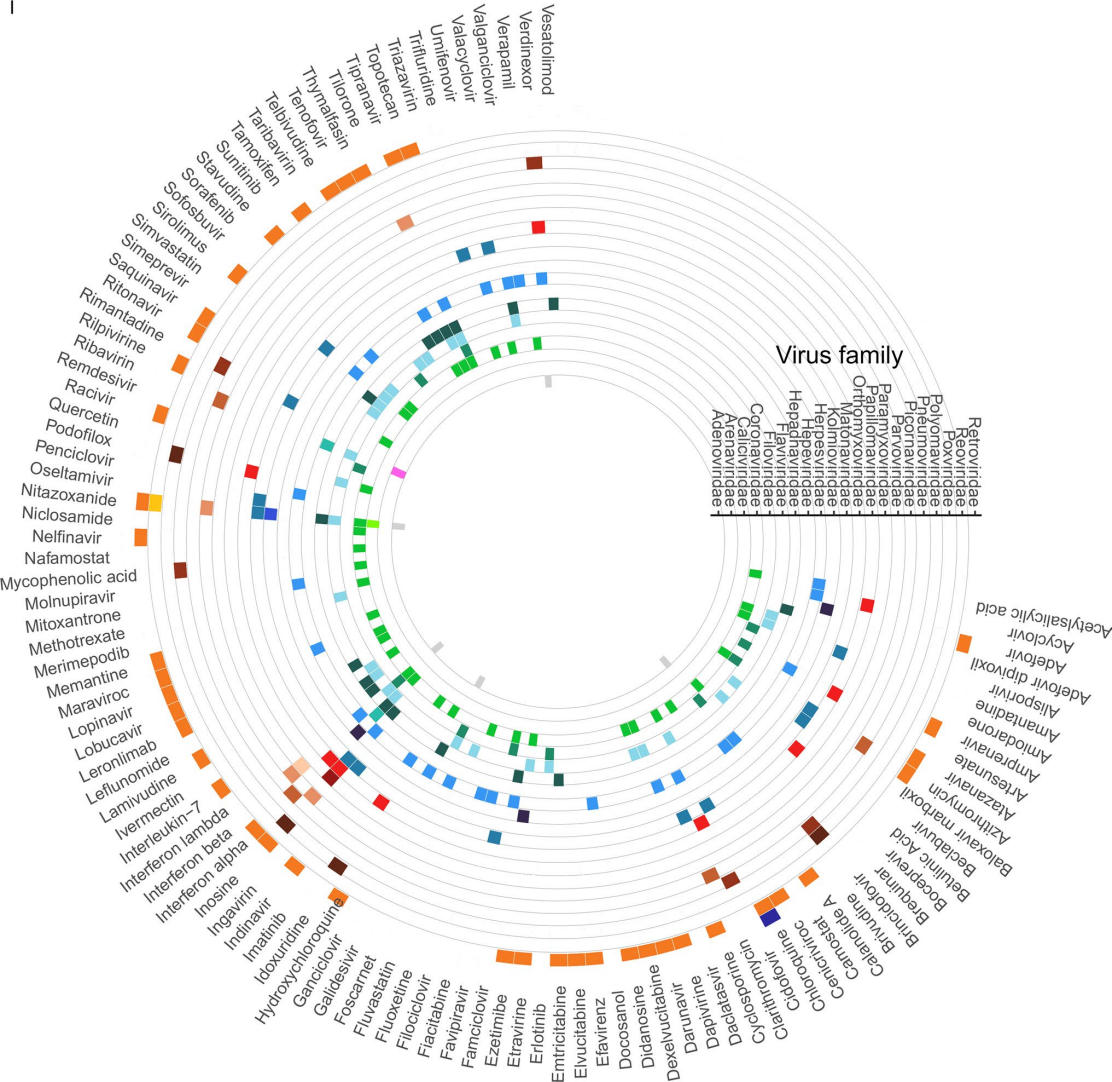
A circular heatmap depicting investigational/approved broad-spectrum antiviral agents (BSAs) and targeted viruses grouped into families

Figure 2 shows a chord diagram depicting the relation between BSAs and virus targets grouped into viral families. The wider connection between viral families the larger the number of BSAs. Interestingly, many BSAs targeting viruses belonging to *Coronaviridae* family also inhibit viruses of *Flaviviridae* family. The figure also indicates that the coverage of viral families by BSAs is far from equal or ideal.

### Combinations of antiviral agents

**Fig. 3:**
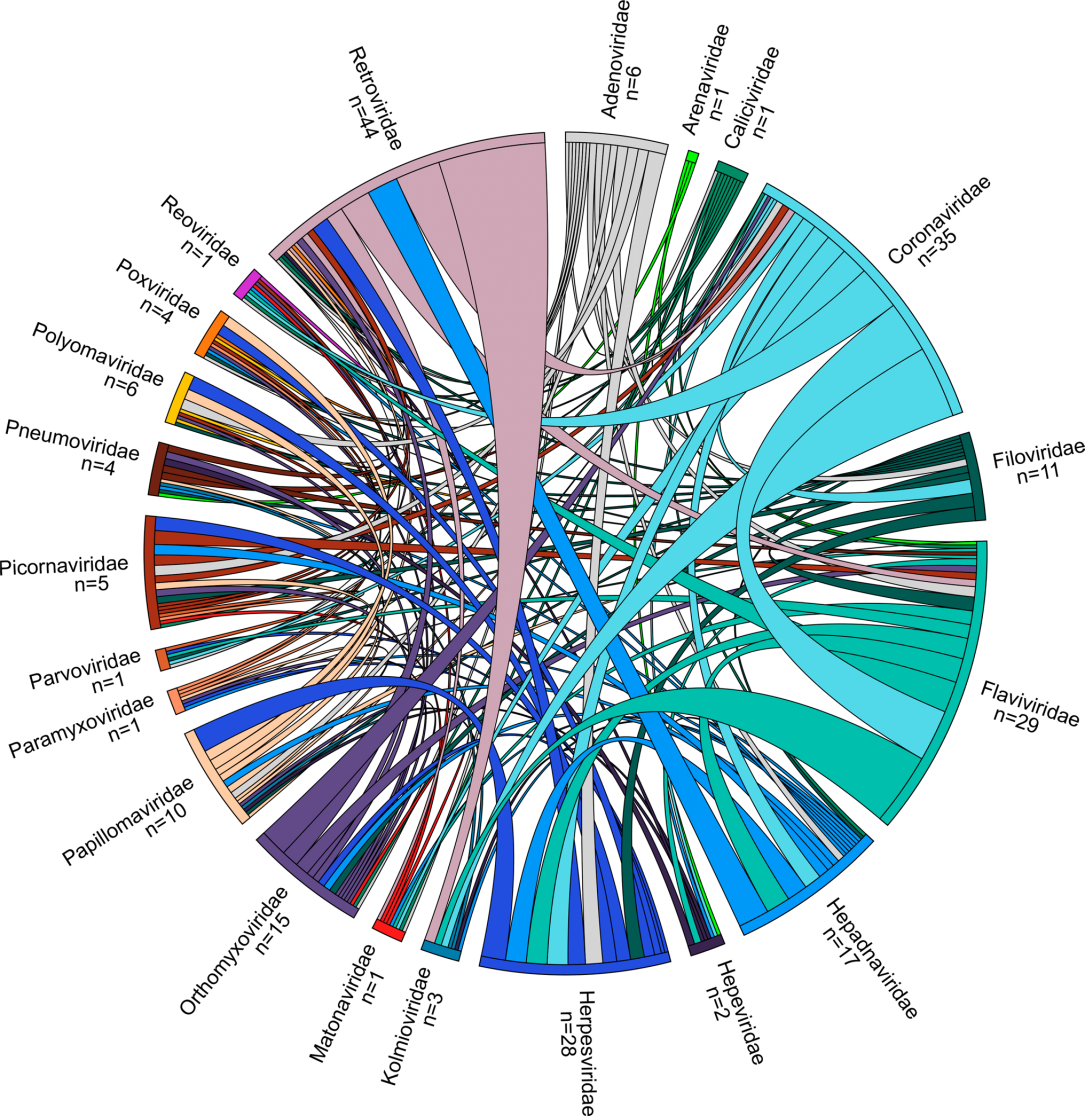
Chord diagram showing the relationship between investigational/approved BSAs and the viruses grouped into families. The wider the lines connecting viral families, the greater the amount of BSAs

**Fig. 4:**
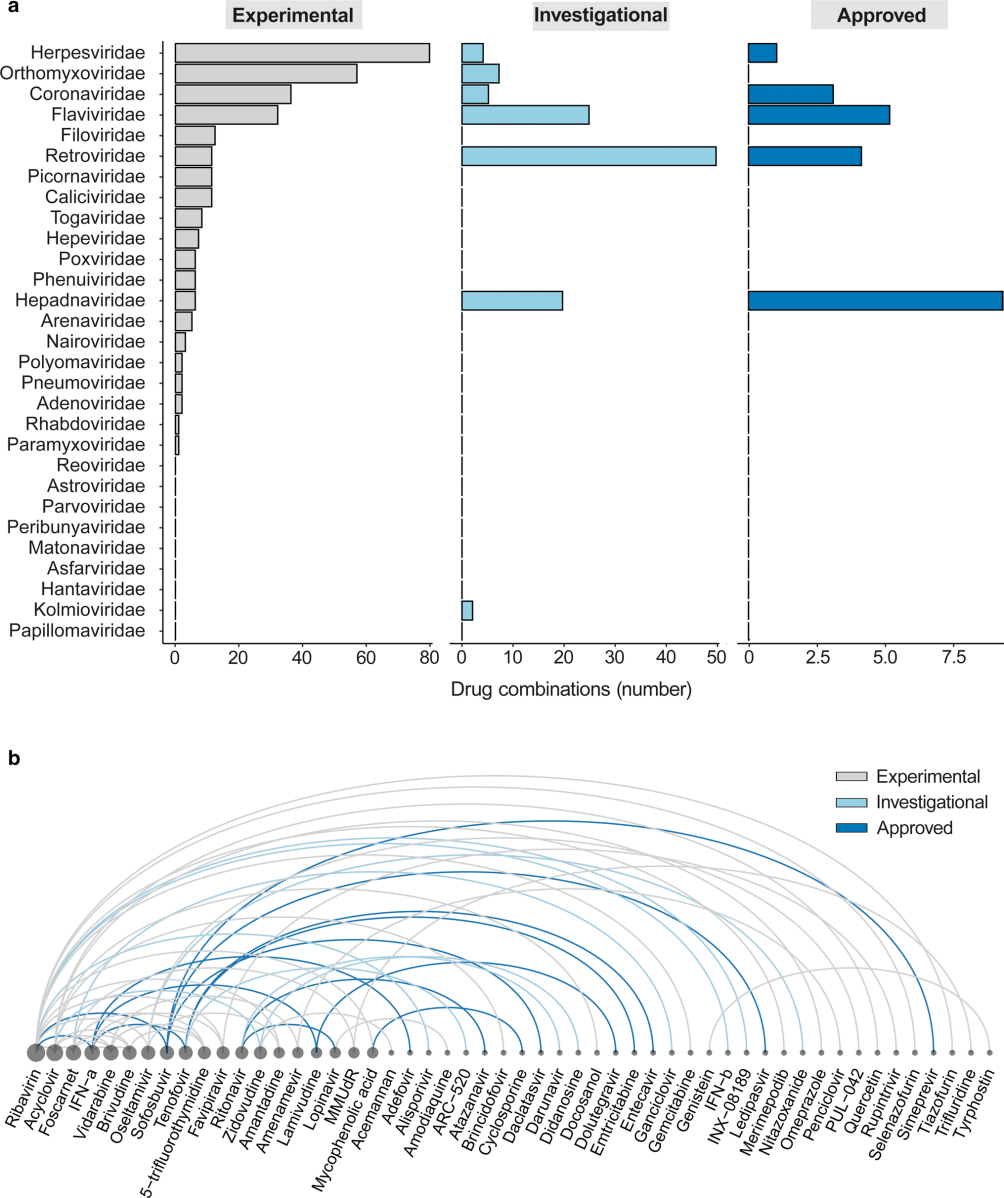
Experimental, investigational, and approved BSA-containing drug combinations (BCCs). **a** A graph shows experimental (gray), investigational (light blue) and approved (blue) BCCs which target viruses of different families. **b** An arc diagram showing examples of BCCs targeting 2 or more viruses. The diagram is ordered according to number of drug combinations that include a particular antiviral compound

To target multiple viruses and mitigate the development of antiviral drug resistance, several antivirals can be administrated simultaneously (Supplementary data) [10, 46, 47]. Synergistic antiviral cocktails contain lower concentrations of agents. Such cocktails may reduce the side effects associated with high doses of monotherapies. The AntiviralCombi.Info web server summarized information on available antiviral drug combinations [10], while DrugVirus.Info server highlighted BSA-containing drug combinations (BCCs; Fig. 3) [5]. DrugVirus.Info database allows tracking the development of antiviral combinations and can be used to identify potential combinations for the treatment of emerging viruses (Fig. 4).

### Antivirals against SARS-CoV-2 infection

Immediately after the SARS-CoV-2 was isolated the virus interactions with host cells were analyzed using systems biology approaches [48]. Researchers identified the critical nodes of the molecular networks (interactomes). Transcriptomics/RNA-sequencing was a key method to accurately detect changes in virus-host interactomes during virus evolution [49]. Several agents (mainly BSAs and Abs) that target the evolutionary conserved nodes and prevent viruses from amplifying within the host have been developed [50]. However, SARS-CoV-2 continue to mutate, reducing the effectiveness of the monotherapies over time. Therefore, synergistic drug cocktails (mainly BCCs) have been tested and shown to mitigate the development of antiviral resistance of virus variants [51, 52]. For example, combinations of IFN-alpha with cycloheximide, camostat, EIDD-2801, remdesivir, or nafamostat were synergistic against SARS-CoV-2 infection in vitro or in vivo [53, 54]. Importantly, these combinations contained lower concentrations of agents than monotherapy and therefore may reduce drug side effects. More recently, antivirals belonging to other classes have been added to the list of anti-SARS-CoV-2 agents. However, many biologics (large molecules) have been shown to induce production of autoantibodies and alter immune response to infection [19, 55–58]. Further research is needed to identify the most efficient and safe anti-SARS-CoV-2 therapeutic option.

## Conclusions

Viruses still infect millions and kill hundreds of thousands of people. To date, about a hundred mono- and combination antiviral therapies have been approved, while thousands are in pre- or clinical development [59, 60]. Here, we reviewed 7 classes of antiviral agents. The information about these agents is scattered across various sources. A single database or resource is needed to accumulate all the information. Such resource containing modes of action can be used to expand the spectrum of activities of available antivirals as well as to develop more effective therapeutics. Also, new methods must be developed to effectively and safely combine antiviral agents to target rapidly evolving viruses. Altogether, these efforts may improve the treatment of viral diseases, leading to a reduction in morbidity and mortality of infected patients. In addition, these efforts are important in preparing for new viral pandemics and epidemics.

## Supplementary Information

Below is the link to the electronic supplementary material.

Supplementary file1 (XLSX 283 KB)

## Data Availability

All data generated or analyzed during this study are included in this published article.
